# Supplementary material for: Clinical characteristics of COVID-19 in older adults. A retrospective study in long-term nursing homes in Catalonia
Source: PLoS One. 2021 Jul 23;16(7):e0255141. doi: 10.1371/journal.pone.0255141 (PMC8301631; doi:10.1371/journal.pone.0255141)
Supplement: Attachment — Submitted filename: Response to reviewers.docx [file pone.0255141.s003.docx]

**RESPONSE TO THE REVIEWERS**

**6. Review Comments to the Author**

**Please use the space provided to explain your answers to the questions above. You may also include additional comments for the author, including concerns about dual publication, research ethics, or publication ethics. (Please upload your review as an attachment if it exceeds 20,000 characters)**

**Reviewer #1: The authors provide to assess the manuscript as suggested by my previous revision. Some minor issues are present:**

**Abbreviations should be written entirely in the first appearance in the text (e.g., COVID-19).**

This item has been fixed in the revised version.

**I suggest modifying the first part of the introduction. In my opinion, the authors should start writing a sentence about what is SARS-CoV-2 before explaining the symptoms caused by it.**

In this update, this part has been addressed with a new introductory text.

**The figures are missing in this new version of the manuscript. Furthermore, there is no references in the text for the figures. I suggest adding them.**

This item has been fixed in the revised version and the figures added.
